# Diagnostic Value of Systemic Inflammatory Response Index for Catheter-Related Bloodstream Infection in Patients Undergoing Haemodialysis

**DOI:** 10.1155/2022/7453354

**Published:** 2022-01-29

**Authors:** Jiajia Yang, Hongmei Wang, Qing Hua, Jian Wu, Ying Wang

**Affiliations:** ^1^Department of Infection Management, The Affiliated Suzhou Hospital of Nanjing Medical University, Suzhou Municipal Hospital, Gusu School, Nanjing Medical University, Suzhou, China; ^2^Department of Nephrology, The Affiliated Suzhou Hospital of Nanjing Medical University, Suzhou Municipal Hospital, Gusu School, Nanjing Medical University, Suzhou, China; ^3^Department of Clinical Laboratory, The Affiliated Suzhou Hospital of Nanjing Medical University, Suzhou Municipal Hospital, Gusu School, Nanjing Medical University, Suzhou, China

## Abstract

**Objective:**

This study was aimed at investigating the diagnostic value of the neutrophil-to-lymphocyte ratio (NLR), platelet-to-lymphocyte ratio (PLR), C-reactive protein-to-albumin ratio (CAR), and systemic inflammatory response index (SIRI) for catheter-related bloodstream infection (CRBSI) in patients undergoing haemodialysis.

**Methods:**

A total of 296 patients undergoing haemodialysis with vascular access were selected and divided into the infected (58 patients) and uninfected (238 patients) groups. Their aetiological and general characteristics were retrospectively collected. The NLR, PLR, CAR, and SIRI were calculated.

**Results:**

The NLR, PLR, CAR, and SIRI values in the infected group were significantly higher than those in the uninfected group (*P* < 0.05). After the anti-infective treatment, the NLR, PLR, CAR, and SIRI values in patients with CRBSI were significantly decreased (*P* < 0.05). The NLR, CAR, and SIRI showed diagnostic efficacy in patients with CRBSI with cut-off values of 4.485 (area under the curve (AUC) = 0.827, 95%confidence interval (CI) = 0.768–0.887), 0.975 (AUC = 0.836, 95%CI = 0.779–0.892), and 3.390 (AUC = 0.947, 95%CI = 0.919–0.976). The CAR and SIRI values in patients with gram-negative bacterial infection were significantly higher than those with gram-positive bacterial infection (*P* < 0.05). The AUCs of CAR and SIRI were 0.693 (0.537–0.848) and 0.821 (0.700–0.942) in differentiating gram-negative and gram-positive bacterial infections, respectively.

**Conclusion:**

Our results showed SIRI as a novel and efficient indicator for the early diagnosis of CRBSI in patients undergoing haemodialysis.

## 1. Introduction

Vascular access is indispensable in haemodialysis [1], with the central venous catheter (CVC) being the most common owing to the following advantages: easy operation, plug and play, high blood flow, and minimal damage. However, as it is an invasive procedure, complications such as deep vein thrombosis, venous stenosis, and catheter-related bloodstream infection (CRBSI) can also occur [2]. Moreover, if CRBSI cannot be timely controlled, some serious complications, such as bacteraemia, brain abscess, endocarditis, and even septic shock, may occur, which greatly increase the patients' mortality [3]. Therefore, early diagnosis and treatment of CRBSI are crucial to improve the prognosis of patients undergoing haemodialysis via the CVC.

Currently, blood culture is the gold standard for the diagnosis of bloodstream infections [4]. However, owing to the long culture cycle and low positive rate of blood culture, patients may miss the best opportunity for diagnosis and treatment. Therefore, if simple, low-cost, and less-invasive routine detection methods such as blood routine can be established, indicators that can be used for the early diagnosis of CRBSI should be determined, which can help prevent and treat CRBSI in patients undergoing haemodialysis, to improve the prognosis. Previous studies have shown that neutrophil-to-lymphocyte ratio (NLR), platelet-to-lymphocyte ratio (PLR), and C-reactive protein-to-albumin ratio (CAR) have certain values for the early diagnosis of nosocomial infection [5–7]. Nevertheless, only a few reports have demonstrated the significance of these indicators in the early diagnosis of CRBSI in patients undergoing haemodialysis. Furthermore, the systemic inflammatory response index (SIRI), which combines the absolute values of neutrophils, monocytes, and lymphocytes, is a novel inflammatory index and has been widely considered in disease diagnosis and prognosis evaluation in recent years [8–11].

Owing to different types of bacterial infections, the treatment methods commonly varied [12, 13]. Therefore, finding molecular indicators for the early identification of different types of bacterial infections will help clinicians choose anti-infective treatment options. This study is aimed at exploring the NLR, PLR, CAR, and SIRI values in the early diagnosis and bacterial-type identification of CRBSI in patients undergoing haemodialysis to provide a reference for the diagnosis and treatment of CRBSI.

## 2. Patients and Methods

### 2.1. Study Patients

From January 2015 to June 2021, a total of 296 patients undergoing haemodialysis using CVCs to establish vascular access in the Blood Purification Centre of Suzhou Hospital Affiliated with Nanjing Medical University were retrospectively selected as study patients. The patients were divided into the infected (58) and uninfected (238) groups based on the CRBSI development.

Patients who met the following criteria were included: (1) the CRBSI diagnosed based on the relevant diagnostic criteria issued by the US Centres for Disease Control in 2008, (2) aged >18 years, and (3) Seldinger technique for deep venipuncture catheterisation.

Patients with the following conditions were excluded: (1) skin infection before catheterisation; (2) complications such as serious heart-, brain-, or liver-related diseases; (3) other types of infections; and (4) incomplete data on clinical and laboratory indicators or nosocomial infection aetiology. Among the 296 patients, 160 were men and 136 were women, with ages ranging from 35 to 79 years, with an average age of 48.64 ± 9.13 years.

All procedures in this study were following the Declaration of Helsinki. This study was approved by the hospital ethics committee (approval number: KL901167).

### 2.2. Collection of Infection Status and Clinical Data

By reviewing the medical records of patients and hospital infection monitoring system, the occurrence and distribution characteristics of the pathogens were retrospectively collected. Furthermore, the patients' age, sex, primary disease, catheterisation site, catheter type, catheterisation days, dialysis time, and whether to use immunosuppressive agents were recorded.

### 2.3. Collection of Laboratory Indicators

By searching the patients' medical records and laboratory forms, indicators including neutrophils (N), lymphocytes (L), platelets (PLT), monocytes (M) counts, C-reactive protein (CRP), and albumin were collected. Then, the NLR, PLR, CAR, and SIRI were calculated. The SIRI was calculated as follows: *N* × *M*/*L*. The laboratory test values within 48 h before the blood culture and 2 weeks after the anti-infective treatment were recorded in patients with CRBSI, whereas the test values were taken on admission in those without CRBSI.

### 2.4. Statistical Analysis

The SPSS 22.0 was used for all statistical analyses. Quantitative variables with normal distribution were described as mean and standard deviation (SD) and compared using the *t*-test. Qualitative data were described as frequency and percentage and compared using the chi-square test. The receiver operating characteristic (ROC) curve was used to evaluate the efficacy of inflammatory composite indices in the early diagnosis of CRBSI in patients undergoing haemodialysis. *P* < 0.05 was considered statistically significant.

## 3. Results

### 3.1. Aetiological and General Characteristics

CRBSI occurred in 58 of 296 patients undergoing haemodialysis, with an infection rate of 19.59%. Using the samples of the 58 infected patients, 67 strains of pathogenic bacteria were cultured, including 40 gram-negative bacteria (59.70%), 23 gram-positive bacteria (34.32%), and 4 fungi (5.97%) (Table 1).

Differences in the catheter-type distribution were observed between the infected and uninfected groups, and the proportion of patients with temporary catheters in the infected group was significantly higher than that in the uninfected group (*P* < 0.05), although no significant differences were found in age, sex, protopathy, catheterisation site, catheterisation days, dialysis time, and use of immunosuppressive agents between the two groups (*P* > 0.05). The results are demonstrated in Table 2.

### 3.2. Comparison of NLR, PLR, CAR, and SIRI between Patients with and without CRBSI

**Figure 1:**
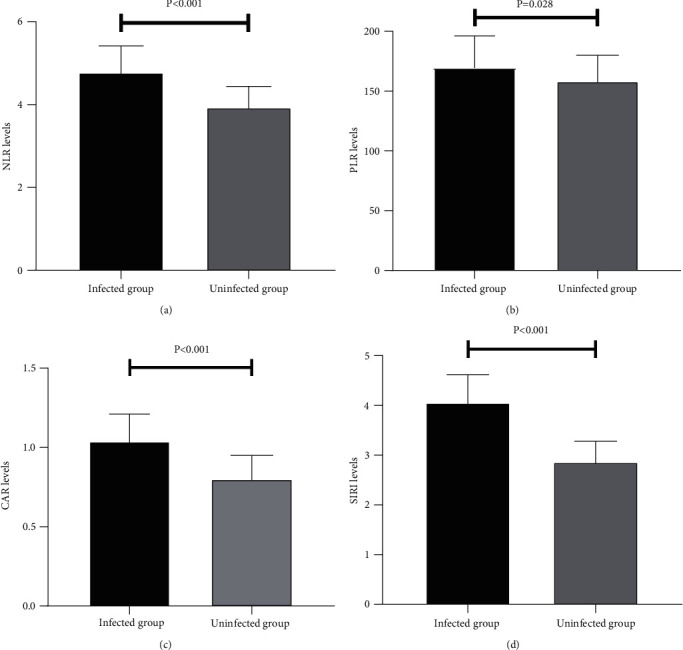
Comparison of inflammatory index values between the infected and uninfected groups: (a) comparison of NLR values; (b) comparison of PLR values; (c) comparison of CAR values; (d) comparison of SIRI values. NLR: neutrophil-to-lymphocyte ratio; PLR: platelet-to-lymphocyte ratio; CAR: C-reactive protein-to-albumin ratio; SIRI: systemic inflammatory response index.

The NLR, PLR, CAR, and SIRI values in the infected group were 4.77 ± 0.65, 169.50 ± 26.70, 1.04 ± 0.17, and 4.06 ± 0.56, respectively, which were significantly higher than those in the uninfected group (NLR, PLR, CAR, and SIRI were 3.93 ± 0.51, 158.14 ± 21.90, 0.80 ± 0.15, and 2.86 ± 0.42, respectively, all *P* < 0.05) (Figure 1).

### 3.3. Comparison of NLR, PLR, CAR, and SIRI in Patients with CRBSI before and after Anti-Infective Treatment

**Figure 2:**
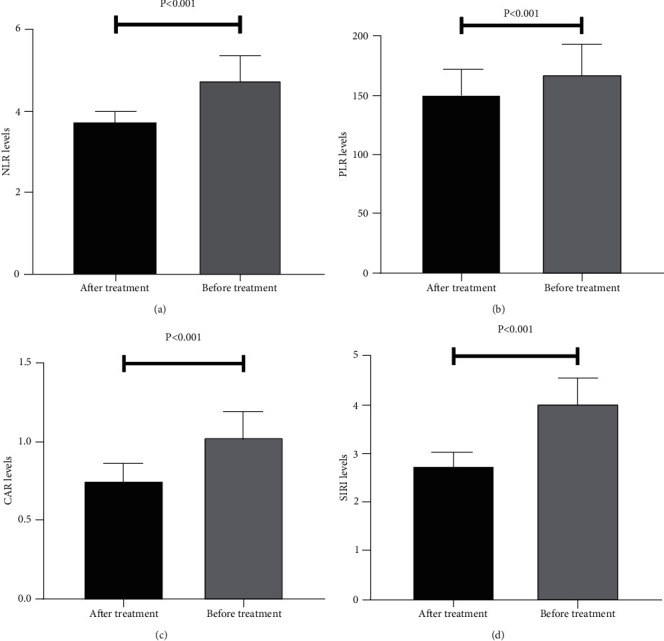
Comparison of NLR, PLR, CAR, and SIRI values before and after anti-infective treatment: (a) comparison of NLR values; (b) comparison of PLR values; (c) comparison of CAR values; (d) comparison of SIRI values. NLR: neutrophil-to-lymphocyte ratio; PLR: platelet-to-lymphocyte ratio; CAR: C-reactive protein-to-albumin ratio; SIRI: systemic inflammatory response index.

After an anti-infective treatment, the NLR, PLR, CAR, and SIRI values in patients with CRBSI were 3.75 ± 0.31, 152.32 ± 22.13, 0.76 ± 0.12, and 2.78 ± 0.31, respectively, which were significantly lower than those at pretreatment (all *P* < 0.001) (Figure 2).

### 3.4. ROC Curve Analysis of NLR, PLR, CAR, and SIRI for the Early Diagnosis of CRBSI

**Figure 3:**
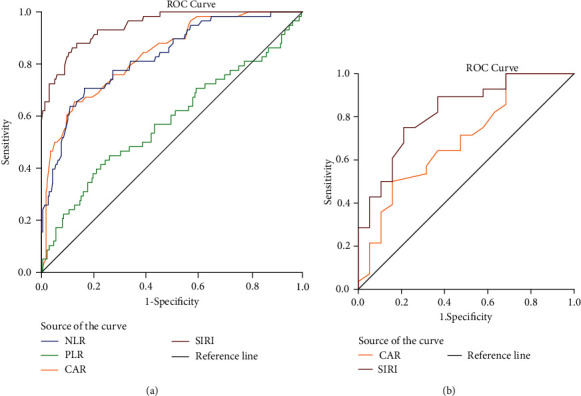
ROC curve of the inflammatory composite index for diagnosing CRBSI and identifying different bacterial types: (a) ROC curves for NLR, PLR, CAR, and SIRI to diagnose CRBSI; (b) ROC curves for CAR and SIRI to distinguish gram-negative and gram-positive bacterial infections. ROC: receiver operator characteristic; NLR: neutrophil-to-lymphocyte ratio; PLR: platelet-to-lymphocyte ratio; CAR: C-reactive protein-to-albumin ratio; SIRI: systemic inflammatory response index; CRBSI: catheter-related bloodstream infection.

To determine the availability of NLR, PLR, CAR, and SIRI for the early diagnosis of CRBSI, ROC curve analysis, a broadly appreciated objective statistical method, was performed. Whether CRBSI developed in patients undergoing haemodialysis was taken as the variable state (no = 0, yes = 1), and NLR, PLR, CAR, and SIRI were considered as test variables, respectively. Finally, except the PLR, the NLR, CAR, and SIRI were effective diagnostic markers in patients with CRBSI with cut-off values of 4.485 (area under the curve (AUC) = 0.827, 95%CI = 0.768–0.887, sensitivity = 70.69%, and specificity = 83.61%), 0.975 (AUC = 0.836, 95%CI = 0.779–0.892, sensitivity = 65.51%, and specificity = 87.39%), and 3.390 (AUC = 0.947, 95%CI = 0.919–0.976, sensitivity = 87.93%, and specificity = 86.56%) (Figure 3(a)).

### 3.5. NLR, PLR, CAR, and SIRI Values in Differentiating Patients with Blood-Cultured Gram-Negative (G^−^) and Gram-Positive (G^+^) Bacteria

**Figure 4:**
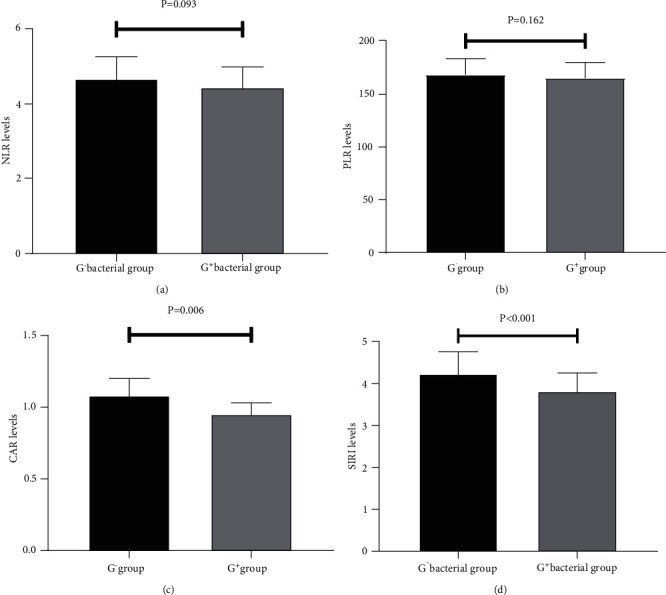
Comparison of NLR, PLR, CAR, and SIRI values between gram-negative and gram-positive groups: (a) comparison of NLR values; (b) comparison of PLR values; (c) comparison of CAR values; (d) comparison of SIRI values. NLR: neutrophil-to-lymphocyte ratio; PLR: platelet-to-lymphocyte ratio; CAR: C-reactive protein-to-albumin ratio; SIRI: systemic inflammatory response index.

The blood culture results indicated that 47 of 58 patients with CRBSI were single bacterial infections, including 28 G^−^ bacterial infections and 19 G^+^ bacterial infections. The NLR, PLR, CAR, and SIRI values in the G^−^ group were 4.72 ± 0.62, 168.71 ± 15.24, 1.08 ± 0.12, and 4.23 ± 0.52, respectively, and those in the G+ group were 4.48 ± 0.59, 165.42 ± 14.86, 0.95 ± 0.08, and 3.81 ± 0.44, respectively. Comparison results between the two groups showed that CAR and SIRI values in the G^−^ group were significantly higher than those in the G^+^ group (*P* < 0.05), whereas NLR and PLR values showed no statistical significance between the two groups (*P* > 0.05) (Figure 4).

ROC curve analysis was performed to evaluate whether CAR or SIRI could be used to identify different bacterial types. The bacterial type was considered as the state variable (G^+^ = 0, G^−^ = 1), and CAR and SIRI were regarded as test variables. CAR and SIRI showed diagnostic values in G^−^ bacterial infection with cut-off values of 0.775 (AUC = 0.693, 95%CI = 0.537–0.848, sensitivity = 50.00%, and specificity = 84.21%) and 2.585 (AUC = 0.821, 95%CI = 0.700–0.942, sensitivity = 75.00%, and specificity = 78.95%) (Figure 3(b)).

## 4. Discussion

CRBSI is the most clinically relevant infection owing to the potential progression to sepsis and death [14]. If the treatment for CRBSI is untimely, it will enormously endanger the health of patients and increase the economic burden on their families [15]. Blood culture is currently the gold standard to diagnose bloodstream infection. However, due to the long culture cycle and low positive rate of blood culture, patients may miss the optimal opportunity for the treatment if relying on blood culture alone [16]. Therefore, a rapid and simple indicator is urgently needed for the early diagnosis of bloodstream infection. This study discussed the application of NLR, PLR, CAR, and SIRI in the early diagnosis of patients with CRBSI.

Previous studies have shown that the incidence of CRBSI in patients undergoing maintenance haemodialysis is approximately 24% [17]. The study results showed that the incidence of CRBSI in patients undergoing haemodialysis was 19.59%, which was slightly lower than that reported by Fram et al. [18]. Gram-negative bacteria were the main pathogenic bacteria (59.7%), followed by gram-positive bacteria (34.3%) and fungi accounting for the lowest proportion (6.0%). The top five pathogenic bacteria were *Escherichia coli* (47.5%), *Staphylococcus aureus* (39.1%), *Pseudomonas aeruginosa* (25.0%), *Staphylococcus cephalus* (21.7%), and *Enterococcus faecium* (17.4%), all of which were common bacteria on the skin surface and widely occurred on the body surface of the medical staff and patients. In invasive diagnostic and therapeutic procedures, pathogenic bacteria on the skin surface of medical staff and patients are the main source of pathogenic bacteria for CRBSI. Moreover, the immune system of patients undergoing haemodialysis is compromised. Hence, they are predisposed to infection through the catheter route while undergoing treatment or dialysis. Therefore, to reduce infections caused by pathogenic bacteria, standard handwashing and strict adherence to aseptic practices are essential to prevent bacteria from passing from the skin to the bloodstream.

Our study found a significant difference in the distribution of catheter types between patients undergoing haemodialysis with and without CRBSI. The proportion of patients with nontunnelled temporary haemodialysis catheters in the CRBSI group was significantly higher than that in the non-CRBSI group, which may be related to the fact that the temporary catheter more likely slips and without the tunnel to prevent bacterial invasion [19]. Inflammatory indicators in patients undergoing haemodialysis were retrospectively collected and found that NLR, PLR, CAR, and SIRI were all significantly increased in patients with CRBSI than those without. Furthermore, the results suggested that NLR, PLR, CAR, and SIRI values after an anti-infective treatment were significantly lower than at pretreatment. Therefore, we assumed that these indicators may be applied in the early diagnosis of CRBSI to a certain degree.

Results of ROC analysis demonstrated that SIRI has decent diagnostic efficiency, with sensitivity and specificity of 87.9% and 86.6%, respectively. Nevertheless, the application of NLR and CAR in the diagnosis of CRBSI has some limitations because of moderate sensitivity or specificity. Therefore, applying SIRI for the early diagnosis of CRBSI is conducive to the timely targeted treatment, which can improve the prognosis of patients. Wu et al. [5] found that NLR had a certain early diagnostic value for bloodstream infection in patients in the emergency department, but its diagnostic sensitivity and specificity were not high. In this study, NLR had good specificity (83.6%) to diagnose CRBSI, whereas its sensitivity was only 70.7%. Hence, using NLR alone to diagnose CRBSI may increase the rate of misdiagnosis.

Fang et al. [20] showed that compared with patients undergoing peritoneal dialysis without infection, NLR and PLR values in patients with infections were significantly higher. Additionally, although the efficacy of NLR and PLR alone in diagnosing peritoneal dialysis-associated infection was not that good, the diagnostic sensitivity and specificity of NLR combined with PLR were pleasing and better than that of high-sensitivity C-reactive protein. Yigit et al. [21] showed that CAR had a high predictive value in periprosthetic infection after total joint arthroplasty; however, the results of our study indicated that the CAR was not satisfactory for the early diagnosis of CRBSI, which may be because of the different types of infection. SIRI is a new compound inflammatory biomarker based on traditional inflammatory cells, including N, L, and M, which can comprehensively reflect the inflammatory state of the body.

Recent studies have demonstrated the diagnostic value of some other markers of bloodstream infection. Shokripour and Omidifar [22] reported that serum procalcitonin, but not CRP, was statistically significant in distinguishing bloodstream infections in patients with malignancy. However, the sensitivity and specificity were approximately 61.52% and 80.08%; hence, the diagnostic efficiency of serum procalcitonin in diagnosing bloodstream infections is mediocre. Chen et al. [23] demonstrated that CRP and serum procalcitonin were effective for the early diagnosis of G^−^ bacterial bloodstream infection with a sensitivity of 71.4% and 64.3% and specificity of 96.2% and 80.8%, respectively. Although the specificities were outstanding, the sensitivities were ordinary. In our study, SIRI was found to have favourable diagnostic efficiency, with sensitivity and specificity of 87.9% and 86.6%, respectively. Therefore, SIRI was more accurate than previously reported markers for bloodstream infections.

Furthermore, owing to its convenience and economy, SIRI plays an important role in predicting the occurrence, development, and prognosis of various diseases. Topkan et al. [14] have found that SIRI could be a novel, sound, and independent predictor of survival outcomes in patients with newly diagnosed glioblastoma multiforme who underwent postoperative Stupp protocol. Chen et al. [24] have demonstrated that SIRI was an effective inflammatory marker to estimate hyperuricaemia in rural Chinese women and was likely to optimise the risk stratification and prevention of hyperuricaemia. A recent study [25] has shown that SIRI was significantly higher in patients with coronavirus disease 2019 (COVID-19) than those in the control group, whereas its diagnostic value in COVID-19 was moderate. In this study, SIRI was used for the early diagnosis of CRBSI in patients undergoing haemodialysis for the first time, and the diagnostic effect was increasing. Therefore, SIRI could be used as a novel indicator for the early diagnosis of CRBSI in patients undergoing haemodialysis.

Furthermore, the results indicated that CAR and SIRI values in the G^−^ bacteria were significantly higher than those in the G^+^ bacteria, whereas NLR and PLR values were not significantly different between the two groups, indicating that CAR and SIRI may play a role in identifying the types of bacterial infections. Further ROC curve analysis displayed that although CAR and SIRI are statistically significant in differentiating between G^−^ and G^+^ bacterial infections, the effectiveness in practical application is mediocre. Sumardi et al. [26] have found that NLR in G^−^ bacteria was lower than that in G^+^ bacteria in patients with sepsis. A recent study [27] has shown that the sensitivity and specificity of NLR in differentiating bloodstream infections caused by G^−^ and G^+^ bacteria were 73.70% and 53.10%, respectively. However, certain limitations exist in recognising different types of bacteria using these composite inflammatory indicators. In the future, further studies on valuable molecular markers for the early identification of different types of bacterial infections are needed.

The current study demonstrated several potential weaknesses. First, all patients included in this study were from the same hospital; therefore, selection bias was inevitable. Second, this study found that the type of catheter was also an influencing factor in the CRBSI development in patients undergoing haemodialysis. However, subgroup analyses were not performed for different types of catheters. Finally, we did not dynamically monitor the NLR, PLR, CAR, and SIRI values of patients with CRBSI pre-, during, and post-infection. In future studies, these aspects will be evaluated when manpower and material resources allow.

## 5. Conclusion

The outcomes of our current retrospective study indicated that NLR, PLR, CAR, and SIRI were higher in patients with CRBSI than those without. SIRI can be used as a novel indicator for the early diagnosis of CRBSI in patients undergoing haemodialysis.

## Figures and Tables

**Table 1:** Distribution characteristics of CRBSI pathogens.

Pathogen type	Number	Proportion (%)
Gram-negative bacteria (G^−^)	40	
Escherichia coli	19	47.50
Pseudomonas aeruginosa	10	25.00
Stenotrophomonas maltophilia	3	7.50
Klebsiella pneumoniae	2	5.00
Acinetobacter baumannii	2	5.00
Acinetobacter jonesi	1	2.50
Others	3	7.50
Gram positive bacteria (G^+^)	23	
Staphylococcus aureus	9	39.13
Staphylococcus capitis	5	21.73
Excrement enterococcus	4	17.39
Staphylococcus epidermidis	2	8.70
Dung enterococcus	2	8.70
Staphylococcus haemolyticus	1	4.35
Fungus	4	
Candida albicans	2	50.00
Aspergillus	2	50.00

**Table 2:** The characteristics of patients with and without CRBSI.

Characteristics	*N*	Infected group (*n* = 58)	Noninfected group (*n* = 238)	*t*/*χ*^2^	*P*
Age (years)		49.12 ± 9.93	48.53 ± 8.16	0.833	0.402
Sex (*N*, %)				2.539	0.111
Male	156	36 (62.07)	120 (50.42)		
Female	140	22 (37.93)	118 (49.58)		
Protopathy (*N*, %)				0.781	0.941
Chronic glomerulonephritis	76	14 (24.13)	62 (26.05)		
Diabetic nephropathy	85	17 (29.31)	68 (28.57)		
Hypertensive nephropathy	112	21 (36.21)	91 (38.24)		
Nephrotic syndrome	7	2 (3.45)	5 (2.10)		
Others	16	4 (6.90)	12 (5.04)		
Cathetering site (*N*, %)				0.108	0.743
Jugular vein	230	46 (79.31)	184 (77.31)		
Femoral vein	66	12 (20.69)	54 (22.69)		
Catheter type (*N*, %)				5.466	0.019
Long-term catheter	192	30 (51.72)	162 (68.07)		
Temporary catheter	104	28 (48.28)	76 (31.93)		
Cathetering days (*N*, %)				0.212	0.645
<28 days	181	37 (63.79)	144 (60.50)		
≥28 days	115	21 (36.21)	94 (39.50)		
Dialysis time (*N*, %)				3.744	0.053
<1 years	223	38 (65.52)	185 (77.74)		
≥1 years	73	20 (34.48)	53 (22.26)		
Whether use immunosuppressants (*N*, %)				0.022	0.883
No	269	53 (91.38)	216 (90.76)		
Yes	27	5 (8.62)	22 (9.24)		

## Data Availability

The datasets used and/or analyzed during the present study are available from the corresponding author on reasonable request.

## Abbreviations

CVC: Central venous catheter
CRBSI: Catheterism-related bloodstream infection
NLR: Neutrophil-to-lymphocyte ratio
PLR: Platelet-to-lymphocyte ratio
CAR: C-reactive protein-to-albumin ratio
SIRI: Inflammatory response index
N: Neutrophils
L: Lymphocytes
PLT: Platelets
M: Monocytes
CRP: C-reactive protein
SD: Standard deviation
ROC: Receiver operating characteristic
AUC: Area under the curve
G-: Gram-negative
G+: Gram-positive.
